# *Chromobacterium spp*. mediate their anti-*Plasmodium* activity through secretion of the histone deacetylase inhibitor romidepsin

**DOI:** 10.1038/s41598-018-24296-0

**Published:** 2018-04-18

**Authors:** Raúl G. Saraiva, Callie R. Huitt-Roehl, Abhai Tripathi, Yi-Qiang Cheng, Jürgen Bosch, Craig A. Townsend, George Dimopoulos

**Affiliations:** 1 W. Harry Feinstone Department of Molecular Microbiology and Immunology, Bloomberg School of Public Health, Johns Hopkins University, Baltimore, MD USA; 2 Department of Chemistry, Johns Hopkins University, Baltimore, MD USA; 3 UNT System College of Pharmacy, University of North Texas Health Science Center, Fort Worth, TX USA; 4 Department of Biochemistry and Molecular Biology, Bloomberg School of Public Health, Johns Hopkins University, Baltimore, MD USA; 5 InterRayBio, LLC, Baltimore, Maryland, USA

## Abstract

The *Chromobacterium sp*. Panama bacterium has *in vivo* and *in vitro* anti-*Plasmodium* properties. To assess the nature of the *Chromobacterium*-produced anti-*Plasmodium* factors, chemical partition was conducted by bioassay-guided fractionation where different fractions were assayed for activity against asexual stages of *P. falciparum*. The isolated compounds were further partitioned by reversed-phase FPLC followed by size-exclusion chromatography; high resolution UPLC and ESI/MS data were then collected and revealed that the most active fraction contained a cyclic depsipeptide, which was identified as romidepsin. A pure sample of this FDA-approved HDAC inhibitor allowed us to independently verify this finding, and establish that romidepsin also has potent effect against mosquito stages of the parasite’s life cycle. Genomic comparisons between *C. sp*. Panama and multiple species within the *Chromobacterium* genus further demonstrated a correlation between presence of the gene cluster responsible for romidepsin production and effective antiplasmodial activity. A romidepsin-null *Chromobacterium spp*. mutant loses its anti-*Plasmodium* properties by losing the ability to inhibit *P. falciparum* HDAC activity, and romidepsin is active against resistant parasites to commonly deployed antimalarials. This independent mode of action substantiates exploring a chromobacteria-based approach for malaria transmission-blocking.

## Introduction

In spite of remarkable progress toward its elimination throughout the last decade, malaria remains endemic in 91 countries, with nearly half of the world’s population at risk in 2016 (212,000,000 new cases and 429,000 deaths estimated in 2015)^1^. Containing the spread of malaria is mainly achieved by the deployment of bed nets and insecticide treatments. Poor compliance and resistance, however, hinder the effectiveness of these efforts. In parallel, antimalarial drugs have been instrumental in preventing the most aggressive and lethal forms of the disease caused by *Plasmodium falciparum*. However, due to the limited structural diversity within the chemical scaffolds of current clinically-available drugs and the increased incidence of drug resistance, new antimalarial compounds with novel modes of action must be identified^2–4^.

Bacteria of the genus *Chromobacterium* are Gram-negative β-proteobacteria of the Neisseriaceae family that occur as flagellated rods or cocci in water or soil environments^5^. Originally only comprised of *C. violaceum –* a purple-pigmented bacterium that has been associated with opportunistic infections in humans – the genus has been expanded over the past ten years and now comprises more than 8 fully-characterized species^6–11^. We have recently described the novel *Chromobacterium sp*. Panama, notable for inducing lethality in larvae and adult *Aedes* and *Anopheles* mosquitoes, as well as *in vitro* and *in vivo* antipathogenic activity against the malaria parasite and the dengue virus^12^. These properties render this bacterium an interesting candidate to control both mosquito populations and pathogen transmission, since manipulation of mosquito gut microbiota has proven successful through exposing mosquitoes to bacteria-spiked artificial nectars^13^.

Concerning its anti-*Plasmodium* potential, *C. sp*. Panama was found to render *Anopheles gambiae* more resistant to malaria parasite infection when laboratory-reared mosquitoes were colonized by the bacterium prior to feeding on infectious blood^12^. This anti-*Plasmodium* activity was proven to be mediated by bacteria-produced and secreted metabolites, as *in vitro* assays independent of the mosquito system showed potent activity against asexual and sexual (both gametocytes and ookinetes) stages of the parasite^12^.

The goal of this study is to characterize the antiplasmodial activity of chromobacteria by isolating and characterizing the secreted factor responsible for *Plasmodium* inhibition. For that purpose, we combine *in silico, in vitro* and *in vivo* approaches to compare the anti-*Plasmodium* activity of a multitude of *Chromobacterium* species to conclude that romidepsin, a known histone deacetylase (HDAC) inhibitor, is responsible for the previously observed anti-*Plasmodium* activity. Romidepsin had already been shown to negatively impact *P. falciparum* asexual^14,15^ and sexual^16^ stages *in vitro*; here we further analyze the spectrum of this activity to include mosquito stages of the parasite and discuss potential applications of this discovery.

## Results

### Anti-*Plasmodium* activity of *Chromobacterium sp*. Panama fractionates with romidepsin

We have previously shown the *in vitro* inhibitory effect of the supernatant of *C. sp*. Panama cultures against different stages of the malaria parasite^12^. To understand the nature of this antiplasmodial activity we employed a bioassay-guided fractionation approach by which the presence of active compounds against asexual stages of *P. falciparum* was evaluated following successive rounds of chemical partition and liquid chromatography of the *C. sp*. Panama supernatant. Mass spectrometric analysis was then used to identify compounds within fractions of interest.

**Figure 1:**
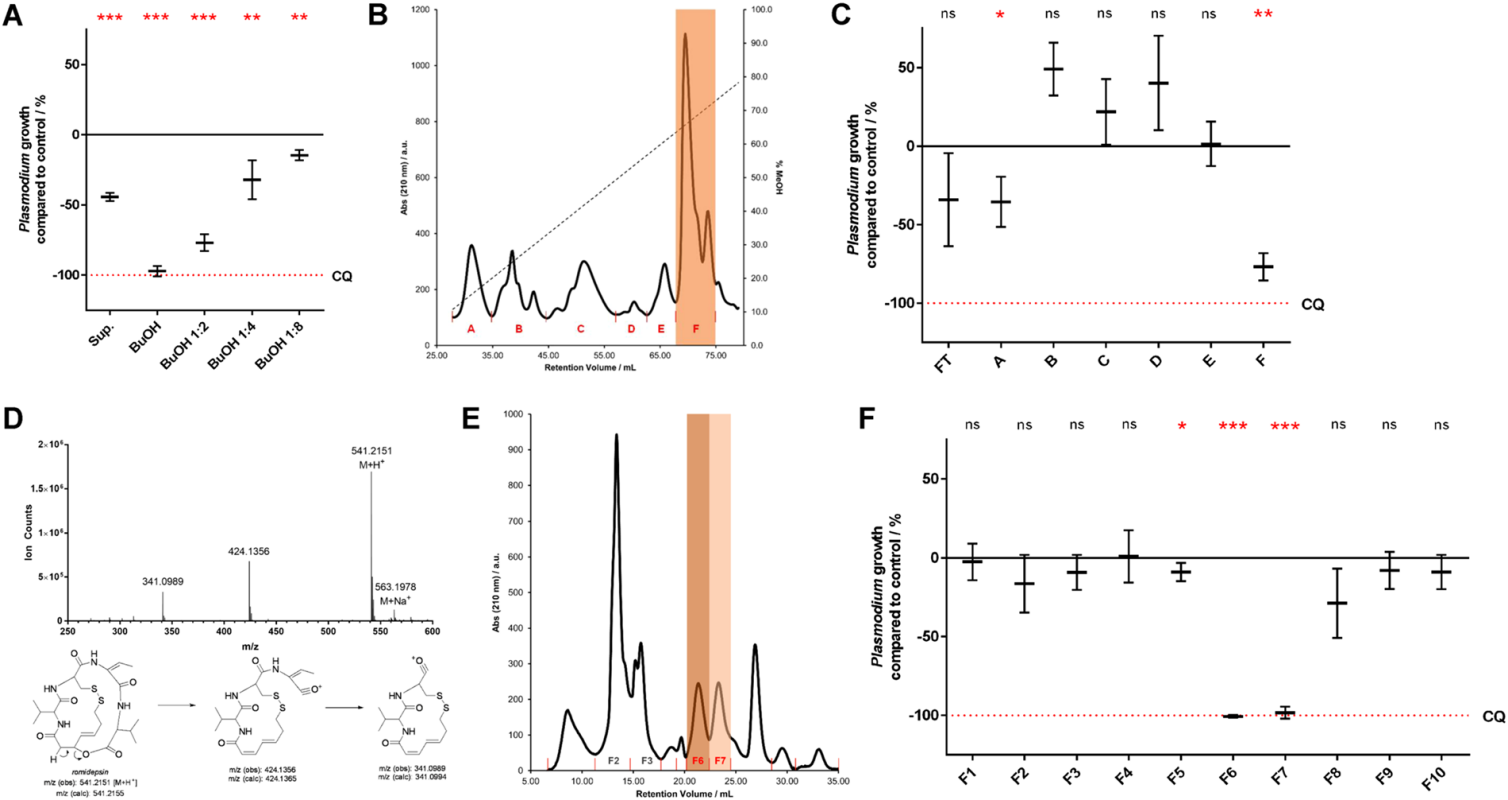
Bioassay-guided fractionation of *Chromobacterium sp. Panama* culture supernatant points to romidepsin as the main antiplasmodial compound. (**A**) Variation in growth of asexual stage *Plasmodium falciparum* NF54 upon incubation with culture supernatant (LB, 72 h, 30 °C) of *C. sp*. Panama, Sup., when compared to that upon incubation with the respective *n*-butanol extract, BuOH and successive 1:2 dilutions. The equivalent of 5 mL of culture was used in each case. 0% inhibition is adjusted for parasite growth in vehicle control (1% DMSO) and 100% inhibition is matched to that of 250 nM chloroquine. Results are shown as mean ± standard deviation of three technical replicates per two biological replicates (total of 6 values); significance was determined using a one-tailed one sample t-test to determine whether each treatment significantly lowered parasite growth compared to control (ns, not significant; *p < 0.05; **p < 0.01; ***p < 0.001). CQ, chloroquine. (**B**) Reverse-phase FPLC chromatogram (absorbance at 210 nm) of *n*-butanol extract of 250 mL of culture supernatant of *C. sp*. Panama (LB, 72 h, 30 °C). Fraction boundaries (A-F) indicated in red; fraction F highlighted. Column: RESOURCE RPC 3 mL. Flow rate: 2 mL/min. Gradient elution with 2%-85% methanol in water 0.1% TFA (dashed line). (**C**) Variation in growth of asexual stage *P. falciparum* NF54 upon incubation with fractions recovered from reverse-phase FPLC (cf. 1B). Fractions were dried and resuspended in proportional amounts of 20% DMSO according to their initial volume. Data presented as 1 A. FT, flow-through. (**D**) Total ion chromatogram from UPLC-ESI-MS analysis of fraction F6. Structures of romidepsin and previously characterized fragmentation products are shown. (**E**) Size exclusion FPLC chromatogram (absorbance at 210 nm) of fraction F (cf. 1B). Fraction boundaries (F1-F10) indicated in red; fractions F6 and F7 highlighted. Column: Superdex Peptide 10/300 GL. Flow rate: 1 mL/min. Isocratic elution with 20% DMSO in water. (**F**) Variation in growth of asexual stage *P. falciparum* NF54 upon incubation with fractions recovered from size-exclusion FPLC (cf. 1E). Fractions processed as 1C; data presented as 1A.

First, the supernatant of a 72 h culture grown in LB medium at 30 °C was subjected to an *n*-butanol-based extraction. This chemical processing retained and concentrated the desired activity against asexual stages of *P. falciparum* NF54 (Fig. 1A), as well as prevented hemolysis that was seldom observed when using the untreated supernatant. Given the enrichment of activity seen after *n*-butanol extraction, we hypothesized that the anti-*Plasmodium* factor was likely to be a relatively lipophilic secondary metabolite, thus informing our next steps.

To identify the suspected small molecule(s) responsible for antiplasmodial activity, the *n*-butanol extract of *C. sp*. Panama was subjected to reversed-phase FPLC, where the extract was partitioned using polystyrene/divinyl benzene as stationary phase and a gradient of methanol in water as mobile phase (Fig. 1B). Collected fractions were dried, resuspended in 20% DMSO and assayed for antiplasmodial activity against *P. falciparum* asexual stages as before. Fraction F was found to contain the most anti-*Plasmodium* activity (Fig. 1C), and thus was carried forward for subsequent analysis.

Higher resolution UPLC separation of fraction F revealed, as expected, that the original crude fraction contained multiple components (Supplementary Fig. S1). Three major components as judged by UV absorption (UPLC) and total ion current (MS) were isolated and designated F-I, F-II and F-III. For F-I, and when sulfur was included as a potential element, the measured mass of 541.2151 gave a predicted elemental composition of C_24_H_37_N_4_O_6_S_2_ with high confidence (Fig. 1D). Two prominent fragment ions were visible indicating sequential losses of *m/z* = 117 and 83, corresponding to fragment losses of –C_5_H_11_NO_2_ and –C_4_H_5_NO, respectively. The observed parent mass and the unusual predicted presence of two sulfur atoms led us to a tentative assignment of the structure to FR901228, or romidepsin, a metabolite previously described from *C. sp*. 968^17^. This structure could be confirmed by unique features of the fragmentation pattern observed in its mass spectrum. Typically, peptide bonds cleave adjacent to the carbonyl to give stable acylium ion fragments. For romidepsin, however, an unusually favorable β-elimination releases the valyl peptide subunit with loss of a proton followed by normal peptide scission to render loss of this amino acid fully intact (Fig. 1D). Sequential loss of a dehydrobutyrine (Dhb, from dehydration of a Thr residue) unit was seen further in accord with the structure assignment to romidepsin^18^.

While romidepsin has a mixed polyketide and non-ribosomal peptide (NRPS) biosynthetic origin, the principal natural products in F-II and F-III had significantly greater molecular weights (1357.6859 and 1195.6311, respectively), but proved to be identical NRPS products that differed only by the presence or absence of a glucose modification. Here the interplay of mass spectrometry, genome sequence information and the availability of *in silico* tools to predict the identity of common amino acid building blocks^19–21^ and their order in a NRPS product allowed us to propose a tentative hexapeptide substructure containing a specific site of *N*-methylation: H_2_N•••Thr–Tyr–Thr–Gln–Gly–*N*-Me-Thr–Xxx(Leu/His/Arg)-COOH. Other fragmentary genomic data pointed to another threonine-activating domain, a putative glycosyltransferase and the presence of a specialized loading domain associated with *N*-terminal acylation by long-chain β-hydroxyacids^22^. High-resolution mass spectrometric observation of the higher mass product, F-II, gave a prominent *m/z* = 1195 fragment consistent with the loss of glucose, followed by a series of fragment ions mirrored precisely in the mass spectrum of F-III. These common fragments allowed the following residue sequence to be assigned: H_2_N-Thr–Tyr–Dbh (from dehydration of Thr)–Gln–Gly–Thr–His-COOH in complete agreement with prediction (Supplementary Fig. S2). The exact masses of F-II and F-III (±glucose) and the unique signature of this shared hexapeptide substructure dictated with high probability that F-II and F-III correspond to the previously investigated antifungal agents Sch 20562 and 20561 (Supplementary Fig. S3)^23,24^.

To further resolve the components of fraction F, orthogonal size-exclusion FPLC (100–7000 g/mol) was employed and showed that this fraction was comprised of at least ten distinct entities as determined by UV absorbance (Fig. 1E). These were individually collected and assayed for antiplasmodial activity against *P. falciparum* asexual stages as before. Potent activity was observed in fractions F6 and F7 (Fig. 1F) and both subfractions gave mass spectrometric data fully consistent with the depsipeptide romidepsin. Why romidepsin elutes as two distinct peaks by size-exclusion FPLC is unclear; it is possible the macrobicyclic structure of romidepsin exists in two distinct structural conformations that are differentiated by a sizing resin, such as its oxidized and reduced forms^25^. Fractions F2 and F3, on the other hand, returned mass signatures consistent with lipodepsipeptides Sch 20561 and Sch 20562; however, no significant antiplasmodial activity was detected from these compounds in our *in vitro* assay against asexual stages of *Plasmodium* (Fig. 1F).

### Antiplasmodial *Chromobacterium spp*. are closely related genetically and encode for romidepsin production

**Figure 2:**
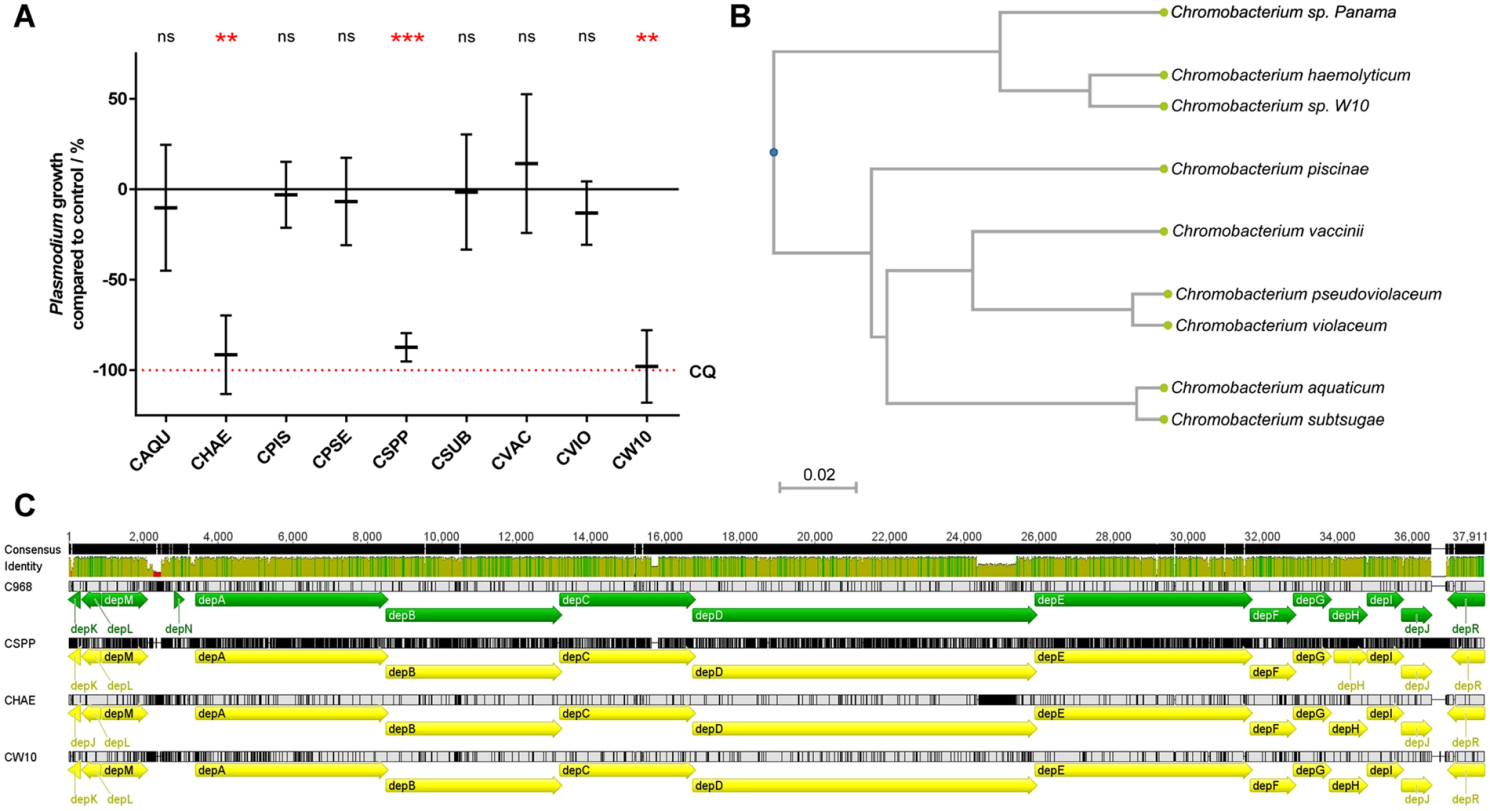
Anti-*Plasmodium* activity of chromobacteria is restricted to the romidepsin-producing subcluster comprised of *C. sp. Panama* and *C. haemolyticum*. (**A**) Variation in growth of asexual stage *P. falciparum* NF54 upon incubation with *n*-butanol extracts of approximately 5 mL of culture supernatants of different *Chromobacterium* species (LB, 72 h, 30 °C). 0% inhibition is adjusted for parasite growth in vehicle control (1% DMSO) and 100% inhibition is matched to that of 250 nM chloroquine. Results are shown as mean ± standard deviation; significance was determined using a one-tailed one sample t-test to determine whether each treatment significantly lowered parasite growth compared to control (ns, not significant; *p < 0.05; **p < 0.01; ***p < 0.001). CQ, chloroquine. CAQU, *C. aquaticum*; CHAE, *C. haemolyticum*; CPIS, *C. piscinae*; CPSE, *C. pseudoviolaceum*; CSPP, *C. sp*. Panama; CSUB, *C. subtsugae*; CVAC, *C. vaccinii*; CVIO, *C. violaceum*; CW10, *C. haemolyticum* W10. (**B**) UPGMA tree generated from a distance matrix inferred from pairwise genome average nucleotide identity (gANI). A distance of 0.02 indicates that the gANI of two species differs by 2 percent points. Cophenetic Correlation Coefficient (CP) = 0.997961877789881. (**C**) Alignment between the reference romidepsin biosynthetic gene cluster in *C. sp*. 968 (C968; GenBank: EF210776.1) to the ones present in the genomes of *C. sp*. Panama (CSPP) and *C. haemolyticum*, both MDA0585 (CHAE) and W10 (CW10) strains. Geneious alignment in Geneious v5.4 (global alignment with free end gaps; cost matrix: 65% similarity).

In parallel with our bioassay-guided fractionation efforts, and to better understand the scope and nature of the anti-*Plasmodium* activity of species belonging to the *Chromobacterium* genus, we obtained a variety of bacterial strains (Table 1) and evaluated their culture supernatants for inhibitory effects on asexual *Plasmodium* stages. Upon parallel *n*-butanol processing of supernatants from 72 h biofilm-forming cultures, our *in vitro* bioassays revealed that antiplasmodial activity was restricted to *C. haemolyticum* (MDA0585 and W10 strains) and *C. sp*. Panama (Fig. 2A). Other species induced a non-significant variation in the growth of *Plasmodium* parasites when compared to vehicle control (1% DMSO), indicating that under our culture conditions only certain species are able to successfully express and secrete the factors responsible for parasite inhibition.

**Table 1:** Bacterial strains in this study.

Species	Strain	Source	Abbreviation	Reference
*C. aquaticum*	CC-SEYA-1	DSMZ (DSM 19852)	CAQU	^7^
*C. haemolyticum*	MDA0585	DSMZ (DSM 19808)	CHAE	^8^
*C. piscinae*	LMG 3947	DSMZ (DSM 23278)	CPIS	^9^
*C. pseudoviolaceum*	LMG 3953	DSMZ (DSM 23279)	CPSE	^9^
*C. subtsugae*	PRAA4-1	DSMZ (DSM 17043)	CSUB	^6^
*C. vaccinii*	MWU205	DSMZ (DSM 25150)	CVAC	^10^
*C. violaceum*	ATCC 12472	ATCC	CVIO	
*Chromobacterium sp*.	968	Yi-Qiang Cheng	C968W	^17^
*Chromobacterium sp*.	968 Δ*depA*	Yi-Qiang Cheng	C968A	^35^
*Chromobacterium sp*.	Panama		CSPP	^67^
*Chromobacterium sp*.	W10	NRRL (B-11053)	CW10	^68^

Next, we sought to understand and differentiate the strains exerting anti-*Plasmodium* effects from those that do not. Having access to genomic information for all species assayed (Table 4), we employed genome average nucleotide identity (gANI) to measure similarities and distances across the entire genomes^26,27^. An UPGMA tree of these results indicated that *C. haemolyticum* and *C. sp*. Panama are closely related and cluster away from the remaining chromobacteria tested (Fig. 2B). This close relationship is consistent with the observation that these two species, and these two alone, seem to carry significant antiplasmodial activity. The distinct clustering of these two species with anti-*Plasmodium* activity suggests that they encode for specific proteins not shared by the other members of the cluster.

To identify the factor(s) responsible for the antiplasmodial activity seen in *C. haemolyticum* and *C. sp*. Panama, the genome of the latter was mined for secondary metabolite biosynthetic gene clusters using antiSMASH^28,29^. It was hypothesized that the anti-*Plasmodium* factor was a secondary metabolite as opposed to a protein, given the enrichment in activity seen after *n*-butanol processing. The retrieved gene clusters were aligned to the genomic sequences of the remaining *Chromobacterium spp*. used in this study and a similarity index for each one was compounded (Table 2; full results are available in Supplementary Table S1). A biosynthetic gene cluster present in both the genomes of *C. haemolyticum* and *C. sp*. Panama and absent from the other chromobacteria was to be considered of special relevance, as it would map with the antiplasmodial activity profile.

**Table 2:** Nucleotide alignment results (megaBLAST) querying putative biosynthetic gene clusters detected in the *Chromobacterium sp. Panama* genome by antiSMASH against the genomes of the remaining *Chromobacterium spp*. used in this study.

Cluster	Type	Source	Size (kb)	megaBLAST similarity index								
				CAQU	CHAE	CPIS		CPSE	CSUB	CVAC	CVIO	CW10
Cluster 01	Homoserine Lactone	antiSMASH	20.7	20%	89%		9%	23%	21%	25%	23%	90%
Cluster 02	Saccharide	ClusterFinder	25.0	65%	86%		66%	66%	65%	60%	70%	86%
Cluster 03	NRPS	antiSMASH	73.0	28%	86%		25%	28%	28%	28%	28%	86%
Cluster 04	Fatty Acid	ClusterFinder	33.7	68%	91%		60%	65%	69%	66%	65%	91%
Cluster 05	NRPS	antiSMASH	45.1	27%	91%		24%	28%	27%	28%	28%	90%
Cluster 06	Terpene	antiSMASH	21.7	31%	93%		31%	27%	31%	24%	27%	84%
Cluster 07	NRPS	antiSMASH	43.2	23%	47%		23%	26%	23%	24%	25%	52%
Cluster 08	Putative	ClusterFinder	15.3	49%	83%		37%	50%	50%	54%	57%	87%
Cluster 09	Putative	ClusterFinder	4.4	52%	88%		54%	39%	51%	54%	39%	80%
Cluster 10	Putative	ClusterFinder	9.5	71%	94%		71%	72%	72%	72%	72%	94%
Cluster 11	Polyunsaturated Fatty Acid/Other Ketosynthase	antiSMASH	51.3	16%	89%		16%	13%	16%	14%	12%	64%
Cluster 12	Putative	ClusterFinder	8.8	39%	93%		43%	40%	39%	43%	43%	92%
Cluster 13	NRPS	antiSMASH	51.5	21%	47%		19%	27%	23%	23%	26%	83%
Cluster 14	Putative	ClusterFinder	7.2	53%	84%		52%	53%	53%	55%	54%	84%
Cluster 15	Bacteriocin	antiSMASH	10.8	28%	42%		28%	28%	28%	28%	23%	72%
Cluster 16	Putative	ClusterFinder	8.5	6%	85%		0%	3%	3%	5%	3%	81%
Cluster 17	Bacteriocin	antiSMASH	10.8	49%	91%		45%	61%	49%	60%	61%	93%
Cluster 18	NRPS	antiSMASH	43.9	34%	86%		33%	27%	34%	29%	30%	83%
Cluster 19	Fatty Acid	ClusterFinder	21.1	74%	92%		75%	76%	74%	75%	76%	92%
Cluster 20	Putative	ClusterFinder	8.9	9%	95%		9%	65%	9%	65%	65%	95%
Cluster 21	Saccharide/NRPS	ClusterFinder	81.2	40%	60%		27%	36%	38%	31%	34%	70%
Cluster 22	Saccharide	ClusterFinder	30.1	30%	33%		28%	29%	30%	31%	28%	33%
Cluster 23	Putative	ClusterFinder	6.5	0%	0%		0%	0%	0%	0%	0%	87%
Cluster 24	Putative	ClusterFinder	4.6	84%	91%		83%	80%	84%	83%	81%	91%
Cluster 25	Putative	ClusterFinder	4.8	74%	89%		66%	78%	74%	74%	79%	91%
Cluster 26	NRPS	antiSMASH	49.6	16%	63%		18%	17%	16%	13%	13%	60%
Cluster 27	Putative	ClusterFinder	8.5	20%	89%		20%	20%	20%	22%	20%	89%
Cluster 28	Putative	ClusterFinder	11.7	13%	22%		0%	6%	6%	13%	6%	21%
Cluster 29	Saccharide	ClusterFinder	31.2	30%	90%		26%	32%	30%	26%	30%	91%
Cluster 30	NRPS	antiSMASH	6.1	0%	4%		0%	2%	0%	2%	0%	92%
Cluster 31	NRPS	antiSMASH	3.1	5%	90%		0%	0%	5%	0%	0%	89%

Similarity index compounded by multiplying query cover with % identity; full results available in Supplementary Table S1. A similarity index of 90% or above indicates that said *C. sp. Panama* gene cluster is present in the genome of that particular species; one of 30% or below indicates with confidence that the cluster is not replicated in the other genome.

Mining of the genome of *C. sp*. Panama allowed for the identification of 31 gene clusters putatively involved in biosynthesis of secondary metabolites. Of these, 5 were revealed to fall within, or proximal to, the similarity index ranges equivalent to their presence in *C. haemolyticum* and absence in the genomes of other chromobacteria (Table 2). Clusters 27 and 31 were discarded from further analysis given their small size (less than 10 kb). Cluster 01 is predicted to encode a homoserine lactone, a known bacterial signaling molecule related to quorum sensing^30,31^. Lack of evidence implicating homoserine lactones as antipathogenic agents led us to discard this cluster from further study. Cluster 03 was extensively analyzed and shown to encode the lipodepsipeptides Sch 20561 and Sch 20562, which had meanwhile been ruled out from having any relevant anti-*Plasmodium* effect (cf. Fig. 1).

Cluster 05, on the other hand, significantly aligned with the biosynthetic gene cluster responsible for the production of romidepsin (Fig. 2C), a previously described anti-tumor drug isolated from *C. sp*. 968^32–34^. Alignment of the original genetic sequence reported for this cluster from *C. sp*. 968 (GenBank: EF210776.1) with those of *C. sp*. Panama and *C. haemolyticum* (both MDA0585 and W10 strains) revealed conservation of the cluster architecture with its 12 genes (*depA*–*depJ*, *depM* and *depR*) being replicated in each sequence (Fig. 2C), except the pseudogene *depN*, which was absent from the new sequences analyzed^35^. Average pairwise sequence identity for coding sequences between the reference *C. sp*. 968 and each of the other species was of 89.6% for CSPP, 98.3% for CHAE and 98.0% for CW10.

### Romidepsin has potent anti-*Plasmodium* activity

Taken together, our bioassay-guided fractionation approach and the genomic analysis of active chromobacteria strongly suggest romidepsin as the source of the antiplasmodial properties of these species. Romidepsin, marketed as Istodax^®^, is a potent histone deacetylase (HDAC) inhibitor and an FDA approved cancer therapeutic against T-cell lymphoma^36–38^. To follow up on our initial findings, we obtained this compound from a commercial source and proceeded to comprehensively study its effects on asexual, sexual and mosquito stages of the parasite.

**Figure 3:**
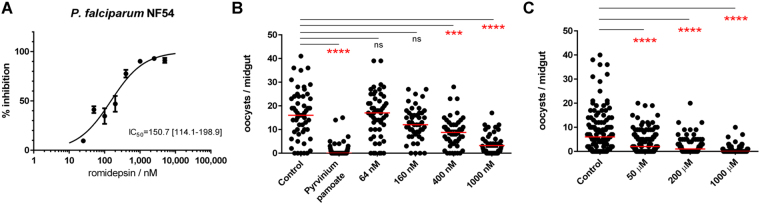
Romidepsin strongly inhibits *Plasmodium*. (**A**) Dose-response curve of romidepsin against *P. falciparum* NF54. 0% inhibition is adjusted for parasite growth in vehicle control (1% DMSO) and 100% inhibition is matched to that of 250 nM chloroquine. IC_50_ is indicated together with the 95% confidence interval returned after fitting a curve to the normalized data using the least squares method with variable slope. (**B**) *P. falciparum* NF54 gametocyte cultures were treated with increasing concentrations of romidepsin, 1 µM pyrvinium pamoate (positive control) or vehicle alone (0.5% DMSO), washed, and fed to *A. gambiae* females. (**C**) Female *An. gambiae* were allowed to feed on romidepsin at the indicated concentrations in 0.5% DMSO 3% sucrose for 1 day prior to being given a *P. falciparum* NF54-infected blood meal; control group was fed on vehicle alone. Oocyst numbers determined 7 days later for two (B) and three (C) independent replicates are shown. Horizontal bars represent the median number of oocysts per treatment. Significance was determined using the Kruskal-Wallis test by comparing treatment groups to the control and correcting for multiple comparisons by the Dunn’s test (ns, not significant; ***p < 0.001; ****p < 0.0001).

As our preliminary assays focused on growth inhibition of asexual *P. falciparum* NF54, we started by replicating these experiments with pure romidepsin, allowing us to control the dose being administered. As shown in Fig. 3A, romidepsin alone exhibits significant activity against this stage of the *Plasmodium* life cycle, with a calculated IC_50_ of 150.7 nM (95% CI: 114.1–198.9 nM). This finding is in agreement with data generated against drug-sensitive and NF54-derived^39^ *P. falciparum* strain 3D7 having an asexual IC_50_ in the 90–140 nM range for this drug, which was reported in the context of screening multiple clinically-approved HDAC inhibitors for anti-parasitic activity^14,15^. Given its potency, we hypothesize that romidepsin is the main antiplasmodial effector of *Chromobacterium spp*., and is unlikely to require other chromobacteria-produced factors for its anti*-Plasmodium* activity.

Next, we investigated the transmission-blocking activity of romidepsin by treating *P. falciparum* NF54 gametocyte cultures with the drug prior to feeding to *A. gambiae* females and evaluating mosquito infection at the oocyst level. Gametocytes were washed ahead of feeding to remove traces of romidepsin, and ensure the observed effects were due to the drug’s activity against early *Plasmodium* sexual stages and not against gametes or other post-fertilization forms; pyrvinium pamoate, a known gametocytocidal compound^40^, was used as positive control. Treatments with 400 and 1000 nM of romidepsin, but not 64 or 160 nM, led to significantly impaired parasite growth within the mosquito (Fig. 3B), which compares to the *in vitro* IC_50_ of 637 nM previously reported against sexual stages of the less-robust gametocyte-producing strain *P. falciparum* 3D7^16^. This indicates that romidepsin has activity against sexual stages of *P. falciparum* and thus can potentially negatively impact transmission.

Our initial findings of antiplasmodial activity of *C. sp*. Panama were seen against mosquito stages of *P. falciparum*. To understand if romidepsin has activity against stages preceding the oocyst in *An. gambiae*, mosquitoes were fed increasing concentrations of romidepsin (vehicle: 0.5% DMSO, 3% sucrose) for 24 h prior to ingestion of a *P. falciparum* NF54 gametocyte-containing blood meal. After 7–8 days post infection, mosquitoes were dissected, and the number of oocysts in each midgut was counted (Fig. 3C). Mosquitoes that were allowed to feed on a 50, 200 or 1,000 µM romidepsin solution were significantly less infected than the control. While these concentrations are greater than the IC_50_ observed *in vitro*, in a 24 h period the mosquitoes will only ingest microliters of the solution^41^, rendering the effective concentration of romidepsin available upon *Plasmodium* infection far lower than that of the original source. Observed variations in infection levels can be explained by variations in the amount of romidepsin ingested by the mosquitoes pre-infection. Furthermore, temporary lethargy was observed in mosquitoes fed at the highest concentrations of the drug, explaining why some failed to complete ingestion of the infected blood meal being, therefore, censored. Mosquito survival upon feeding on the different romidepsin-containing solutions was not affected when compared to control (Supplementary Fig. S4).

### Romidepsin production is required for *Chromobacterium spp*. anti-*Plasmodium* properties

**Figure 4:**
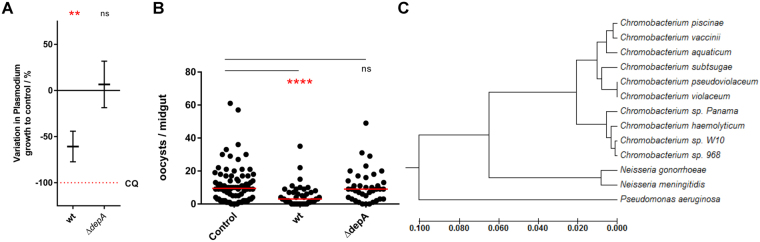
Romidepsin production is necessary and sufficient for the antiplasmodial activity of *Chromobacterium spp*. (**A**) Variation in growth of asexual stage *P. falciparum* NF54 upon incubation with *n*-butanol extracts of approximately 5 mL of culture supernatants (LB, 72 h, 30 °C) of *C. sp*. 968 wildtype (wt) and depA-null mutant (∆*depA*). 0% inhibition is adjusted for parasite growth in vehicle control (1% DMSO) and 100% inhibition is matched to that of 250 nM chloroquine. Results are shown as mean ± standard deviation; significance was determined using a one-tailed one sample t-test to determine whether each treatment significantly lowered parasite growth compared to control (ns, not significant; *p < 0.05; **p < 0.01; ***p < 0.001). CQ, chloroquine. (**B**) Female *An. gambiae* were provided either PBS, *C. sp*. 968 wildtype (wt) or its ∆*depA* mutant suspended within 3% sucrose. After 1 day of being allowed to feed on these suspensions, they were given a *P. falciparum* NF54-infected blood meal, and oocyst numbers were determined 7 days later; oocyst counts for three independent replicates are shown. Horizontal bars represent the median number of oocysts per treatment; inhibition (%) was estimated based on the comparison of these values to that of the PBS control. Prevalence represents the proportion of infected mosquitoes per group. Significance was determined using the Kruskal-Wallis test by comparing treatment groups to the control and correcting for multiple comparisons by the Dunn’s test (ns, not significant; *p < 0.05; **p < 0.01; ***p < 0.001). (**C**) Evolutionary relationships between *C. sp*. 968, other chromobacteria and other related species (*Neisseria gonorrhoeae* NCTC13800; *Neisseria meningitidis* LNP21362; *Pseudomonas aeruginosa* DSM 50071) based on 16 S rRNA genomic sequences as inferred by the UPGMA method. The optimal tree with the sum of branch lengths equal to 0.33018119 is shown. The tree is drawn to scale, with branch lengths in the same units as those of the evolutionary distances used to infer the phylogenetic tree. The analysis was conducted in MEGA6 and involved 13 nucleotide sequences for a total of 925 positions in the final dataset.

Having established that chromobacteria with antiplasmodial properties produce romidepsin, and having demonstrated the ability of romidepsin to inhibit growth and maturation of *P. falciparum in vitro* and *in vivo*, it became essential to understand if romidepsin production was a necessary and sufficient condition for the anti-*Plasmodium* effect seen in supernatants of *Chromobacterium spp*. cultures. For this purpose, *n*-butanol extracts of *C. sp*. 968 and a derived mutant lacking the *depA* gene and thus incapable of secreting romidepsin^35^ were tested against asexual stages of *P. falciparum* as before. The ∆*depA* mutant was found to have no antiplasmodial activity *in vitro* (Fig. 4A).

The romidepsin-null mutant was also fed to *An. gambiae* mosquitoes and its induced lethality did not differ from that of wildtype control (Supplementary Fig. S5), indicating that romidepsin does not appear to be significantly contributing to the mosquitocidal activity previously seen with *C. sp*. Panama against adult *Aedes* and *Anopheles* mosquitoes^12^. Upon infection with *P. falciparum* NF54, mosquitoes that had previously fed on the wildtype or ∆*depA* bacteria exhibited different infection levels: *Plasmodium* maturation was significantly impaired in those that ingested wildtype *C. sp*. 968, whereas those exposed to the romidepsin-null mutant showed no variation compared to the control (Fig. 4B). Of note, the reduction in the number of mosquitoes between the control and the experimental groups is a result of the entomopathogenic properties of these chromobacteria.

These results clearly place *C. sp*. 968 among the *Chromobacterium* species with anti-*Plasmodium* activity. While *C. sp*. 968 was previously believed to be a strain of the *C. violaceum* species^32,42^, upon phylogenetic analysis based on 16 S rRNA gene sequence comparisons it became apparent that this strain clusters with *C. haemolyticum* and *C. sp*. Panama and not with *C. violaceum* (Fig. 4C). This observation is in agreement with our initial description of a branch of chromobacteria around *C. haemolyticum* that exclusively possesses antiplasmodial activity. Furthermore, *C. sp*. 968 colonies are tan, similar to those of *C. haemolyticum* and *C. sp*. Panama, and not purple like *C. violaceum* and other closely related species due to violacein production. These findings and observations point to *C. sp.* 968 not being a *C. violaceum* strain and belonging to a separate cluster of chromobacteria able to produce romidepsin. The absence of a full genomic sequence for the 968 strain precludes us from making a concrete speciation assignment at this point.

### *Chromobacterium spp*. inhibit *P. falciparum* HDAC activity and romidepsin is active against drug resistant isolates

**Figure 5:**
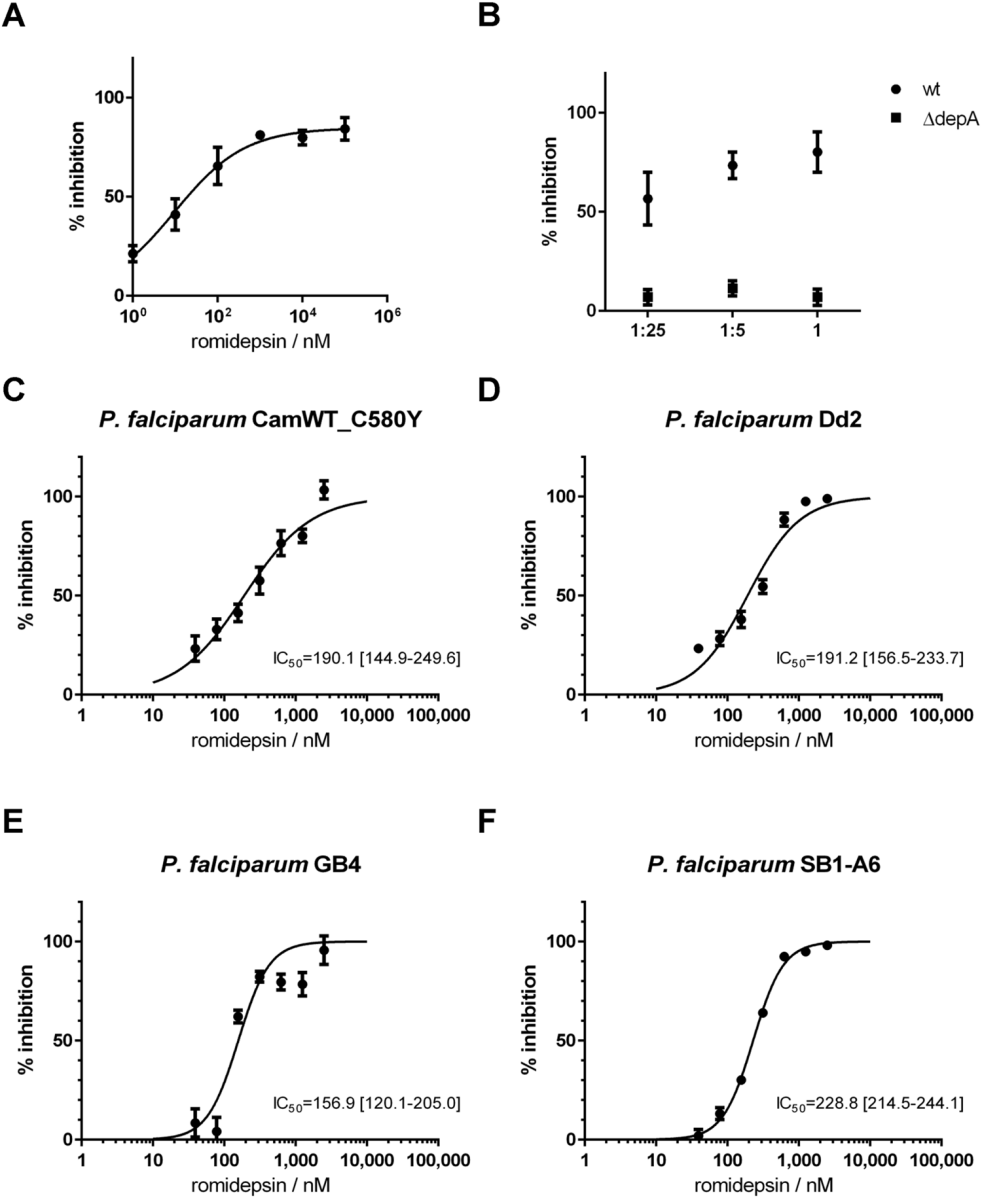
Romidepsin surpasses common drug resistance mechanisms and interferes with *P. falciparum* HDAC activity. Inhibition of HDAC activity of *P. falciparum* NF54 nuclear protein extracts in the presence of (**A**) increasing concentrations of romidepsin or (**B**) *n*-butanol extracts of approximately 5 mL (1×), 1 mL (1:5) and 200 µL (1:25) of culture supernatants (LB, 72 h, 30 °C) of *C. sp*. 968 wildtype (wt) and depA-null mutant (∆*depA*) as measured by endpoint immunodetection of remaining acetylated histone substrate following incubation. Results are normalized to vehicle control of 1.3% DMSO (0% inhibition) and absence of *P. falciparum* nuclear protein extract (100% inhibition). Dose-response curve of romidepsin against *P. falciparum* CamWT_C580Y (**C**, resistant to artemisinin), Dd2 (**D**, resistant to chloroquine, pyrimethamine and mefloquine), GB4 (**E**, resistant to chloroquine) and SB1-A6 (**F**, resistant to cytochrome bc1 inhibitors such as atovaquone). 0% inhibition is adjusted for parasite growth in vehicle control (1% DMSO) and 100% inhibition is matched to that of 250 nM chloroquine. IC_50_ is indicated together with the 95% confidence interval returned after fitting a curve to the normalized data using the least squares method with variable slope.

Romidepsin has been described as an HDAC inhibitor with relevant activity against a *P. falciparum* 3D7 nuclear extract^14^. To test this in our system, we incubated a nuclear protein extract of asexual *P. falciparum* NF54 with an acetylated histone substrate in the presence of the drug. The ratio of remaining acetylated histone substrate in romidepsin-treated *vs*. no-drug control was determined by ELISA after 1 h incubation. Romidepsin suppressed the HDAC activity of the *P. falciparum* nuclear protein extract in a concentration-dependent manner (from 21% inhibition at 1 nM to 80–84% at 1–100 µM; Fig. 5A). Romidepsin has been characterized as a potent inhibitor of human class I HDAC enzymes and a weak inhibitor of class II HDACs, while there is no known activity against class III enzymes^25^. *P. falciparum*, in turn, expresses at least 5 HDAC enzymes: one homologue of class I (PfHDAC1), two of class II (PfHDAC2 and PfHDAC3) and two of class III (PfSir2A and PfSir2B)^43^. A maximum inhibition of ~80% of *P. falciparum* HDAC activity by romidepsin even at high concentrations could be explained by its lack of activity against the class III enzymes together with a potential partial inhibition of the class II HDACs; specific activity against the class I member PfHDAC1 has been previously demonstrated^14^.

Given the results presented thus far, it is expected that a romidepsin-positive culture supernatant extract of wildtype *C. sp*. 968 possesses inhibitory activity against *Plasmodium* HDACs, the contrary being true for a romidepsin-negative extract of its Δ*depA* mutant. To test this hypothesis, *n*-butanol extracts of comparable culture supernatants of these bacterial strains were prepared as before and tested for anti-HDAC activity via ELISA as above. Inhibitory HDAC activity was detected for the wildtype strain extract and its dilutions (from 57% inhibition at 1:25 to 80% undiluted, Fig. 5B), but not for the Δ*depA* extract. This finding further provides a conclusive link between romidepsin-production and anti-*Plasmodium* activity of *Chromobacterium spp*., now substantiated at a mechanistic level.

We then tested the inhibitory activity of romidepsin against *P. falciparum* strains known for being resistant to commonly used antimalarial drugs to understand if romidepsin is able to bypass their resistance mechanisms. These strains included *P. falciparum* CamWT_C580Y, a K13-propeller mutant associated with increased resistance to artemisinin; Dd2, resistant to chloroquine, pyrimethamine and mefloquine; GB4, resistant to chloroquine; and SB1-A6, resistant to inhibitors of cytochrome bc1 electron transport such as atovaquone^44^. Compared to *P. falciparum* NF54, which exhibited an IC_50_ of 150.7 nM (95% CI: 114.1–198.9 nM, Fig. 3A), strains CamWT_C580Y (IC_50_ = 190.1 nM, 95% CI: 114.9–249.6 nM), Dd2 (IC_50_ = 191.2 nM, 95% CI: 156.5–233.7 nM) and GB4 (IC_50_ = 156.9 nM, 95% CI: 120.1–205.0 nM) all showed overlapping intervals for IC_50_ values with 95% confidence (Fig. 5C–E), indicating no significant difference in activity of romidepsin against these drug-resistant variants compared to wildtype. An IC_50_ of 130 ± 40 nM previously reported for romidepsin against *P. falciparum* Dd2^14^ is comparable to our data. Therefore, phenotypic resistance to artemisinin, chloroquine, pyrimethamine and mefloquine does not appear to alter the inhibitory activity of romidepsin towards *P. falciparum*. For *P. falciparum* SB1-A6, the calculated IC_50_ was 228.8 nM (95% CI: 214.5–244.1, Fig. 5F), representing a 1.5 fold increase when compared to that of the NF54 strain. While this was a significant change, the low resistance factor of 1.5 – or 1.08 when taking the closest values in the boundaries of each of the confidence intervals – seems rather negligible and does not provide any strong evidence that the anti-*Plasmodium* activity is hindered when the parasite lacks requirement for electron transport through the cytochrome bc1 complex.

## Discussion

We have previously established that *C. sp*. Panama is able to limit the development of the malaria parasite through colonization of the *An. gambiae* midgut, and that its bacteria-free culture supernatant inhibits *Plasmodium* growth *in vitro*^12^. In the present work, we further probe into this anti-*Plasmodium* activity, showing by bioassay-guided fractionation and multigenomic comparative analysis that romidepsin is the most likely *Chromobacterium*-produced metabolite responsible for its antiplasmodial activity. Using romidepsin obtained from a commercial source, we demonstrate its potent inhibitory effect against asexual, sexual and mosquito stages of the parasite’s life cycle. Furthermore, we validate that a romidepsin-null *Chromobacterium* mutant loses its anti-*Plasmodium* effect both against blood and mosquito stages. As comparable amounts of drug are needed to inhibit resistant variants of *P. falciparum* to commonly deployed antimalarial drugs, romidepsin seems to exert is activity by a distinct mechanism; ours and others *in vitro* measurements show its ability to limit HDAC activity of *P. falciparum* nuclear protein extracts, pointing to its mode of action as an HDAC inhibitor against these parasite enzymes crucial for regulation of gene expression.

Romidepsin was first characterized as an inhibitor of human class I and class II HDAC enzymes and is currently FDA-approved for treatment of T cell lymphoma^36^. Our data indicate that this drug is also potentially effective as an antimalarial with an IC_50_ of 150.7 nM against asexual *P. falciparum* NF54. This finding is in agreement with data generated by others against drug-sensitive *P. falciparum* 3D7^14,15^. For gametocytocidal activity, our data are comparable to that reported also in *P. falciparum* 3D7 in the context of screening for repurposing multiple approved drugs for malaria control^16^. Previous observations showing that treatments with romidepsin led to hyperacetylation of both histone and non-histone *P. falciparum* proteins, and relative inhibition of recombinant PfHDAC1^14^, further reinforce the conclusion that it is the HDAC inhibitory activity of romidepsin that likely underlies its anti-*Plasmodium* effects. In fact, HDAC inhibitors have been pursued as antiparasitic drugs since the discovery in the mid-1990s that the also cyclic tetrapeptide apicidin targets *Plasmodium* and other Apicomplexa by limiting HDAC activity^45^. FR23522, yet another natural cyclic tetrapeptide, has also been described for its anti-apicomplexan properties by targeting TgHDAC3 in *Toxoplasma gondii*^46^, a class I HDAC with homology to PfHDAC1 that shares an Apicomplexa-specific two-residue insertion within the catalytic site of the enzyme^47^.

As is true for these other cyclic tetrapeptides^43^, however, romidepsin does not appear to be an ideal candidate as indicated by others electing to decline performing further tests with this drug in view of its selectivity index^15^: the ratio between effective inhibitory dosages against mammalian and *Plasmodium* targets indicates that there would be an unacceptable level of side-effects if this drug were to be used in its current form^14,15^. The majority of patients experience nausea, vomiting and anorexia, and some progressive fatigue and occasional fever, when undergoing romidepsin regimens^36^. There is, nonetheless, some promise in considering romidepsin as a lead compound for studies to develop a novel drug with increased selectivity for *Plasmodium* HDAC enzymes when compared to their human counterparts, as has been explored with some success for apicidin^48,49^.

While the direct use of romidepsin as a therapeutic drug against malaria cannot be advocated at this time, the same cannot be said when it comes to its transmission-blocking capabilities. Faithful to our original approach of using *Chromobacterium spp*. to suppress parasite infection of the mosquito vector, uncovering romidepsin as the causal agent of its anti-*Plasmodium* effect reinforces the value of this strategy. Control of mosquito populations remains the most widespread and perhaps most valuable strategy for malaria control. As discussed, chromobacteria are able to exert entomopathogenic activity in the malaria vector *An. gambiae* when colonizing their midgut through a mechanism that appears to be independent of their ability to secrete romidepsin (Supplementary Fig. S5). For those mosquitoes that survive this colonization, however, the mechanisms for a second-line control level are now substantiated. *Chromobacterium spp*. will secrete romidepsin and suppress parasite infection in the mosquito, leading to a potential transmission-blocking that would have an epidemiologically significant impact. This compound is shown for the first time to have a significant limiting effect on mosquito stages of the *Plasmodium* life cycle, and it does so by a mechanism distinct from those of currently deployed antimalarials. As such, it is not expected that any pressure applied on the parasite by this HDAC inhibitor as it cycles through the mosquito will result in resistance to any existing antimalarial drug, placing the use of a chromobacteria-based strategy against anopheline mosquitoes as an important additional tool for an integrated approach to malaria control. Further studies currently underway in the semi-field with *Chromobacterium*-spiked attractive sugar baits will determine the viability of this approach.

## Methods

### Ethics Statement

Anonymous commercial human blood (Interstate Blood Bank Inc.) was used for parasite cultures and mosquito feeding, and informed consent was therefore not applicable. The Johns Hopkins School of Public Health Ethics Committee has approved this protocol.

### Bacterial cultures and *n*-butanol extraction

Unless otherwise noted, *Chromobacterium spp*. (Table 1) were grown for 72 h at 30 °C in LB Lennox broth (Sigma, L3022) without agitation, allowing for the formation of a biofilm at the surface of the culture. For *n*-butanol-based extraction, cultures were then thoroughly mixed 1:1 with H_2_O-saturated *n*-butanol and the top organic phase was recovered. Following a short evaporation step under reduced pressure, the mixture was run through a filter (particle retention size of 10 µm) and the filtrate was then evaporated in its entirety and the resulting residue resuspended in methanol. Subsequently, the sample was added to the same volume of 1:1 petroleum ether/diethyl ether under constant agitation, filtered (particle retention size of 1 µm) and the residue obtained resuspended in DMSO and stored at −20 °C until further use. To ensure comparability, cultures of different chromobacteria were run in parallel and equivalent volumes of culture and solvents applied to the chemical extraction protocol.

### *Plasmodium falciparum* strains and cultivation

*P. falciparum* strains (Table 3) were maintained in continuous culture according to the method described by Trager and Jensen^50^. Briefly, *P. falciparum* was grown in O + red blood cells at 2% hematocrit and RPMI 1640 medium supplemented with 10 mM glutamine, 25 mM HEPES, 50 µg/ml hypoxanthine and 10% O + human serum. In order to ensure a microaerophilic environment, the parasites were maintained in a candle jar at 37 °C. Use of human erythrocytes to support the growth of *P. falciparum* was approved by the Internal Review Board of the Johns Hopkins University Bloomberg School of Public Health.

**Table 3:** *Plasmodium falciparum* strains in this study.

Strain	Source: BEI Resources	Resistance traits	IC_50_/nM
CamWT_C580Y	MRA-1251	artemisinin	ND
Dd2	MRA-150	chloroquine, pyrimethamine, mefloquine	87.8 [41.6–185.5] (chloroquine)
GB4	MRA-925	chloroquine	>12,500 (chloroquine)
NF54	MRA-1000	drug sensitive	7.8 [1.8–34.4] (chloroquine)
SB1-A6	MRA-1002	cytochrome bc1 inhibitors (*e.g*. atovaquone)^44^	4065 [888–18613] (atovaquone)

IC_50_ for a representative drug is indicated together with the 95% confidence interval returned after fitting a curve to the normalized data using the least squares method with variable slope.

### Fast Performance Liquid Chromatography

FPLC was performed using an ÄKTA Explorer system. Reverse-phase FPLC was conducted on the *n*-butanol extract of *C. sp*. Panama (resuspended in start buffer) using a RESOURCE RPC 3 mL (GE Healthcare Life Sciences) column under gradient elution between 2% and 85% methanol in water 0.1% TFA at a constant 2 mL/min flow rate. Fraction F was collected, dried under vacuum, and the resulting residue resuspended in 20% DMSO. Size exclusion FPLC of this sample was performed on a Superdex Peptide 10/300 GL (GE Healthcare Life Sciences) column under isocratic elution with 20% DMSO in water at a constant flow rate of 1 mL/min.

### *Plasmodium* asexual stage growth inhibition assay

Antiplasmodial activity of *Chromobacterium spp*. bacterial culture extracts against asexual stages of *P. falciparum* was assessed using a SYBR green I-based fluorescence assay as described earlier^51,52^. Different concentrations of filtered bacterial culture extracts, their fractions or pure compound in 20% DMSO were dispensed in triplicate wells of 96 well microplates, followed by addition of synchronous ring stage *P. falciparum* cultures at 1% hematocrit and 1% parasitemia; parasites were synchronized using 5% Sorbitol as described previously^53^. Chloroquine (250 nM) was used as positive control and 1% DMSO (i.e. final DMSO concentration) was used as negative control. After 72 h of incubation in a candle jar at 37 °C, equal volume of SYBR green-I solution (Invitrogen) in lysis buffer [Tris (20 mM; pH 7.5), EDTA (5 mM), saponin (0.008%; w/v) and Triton X-100 (0.08%; v/v)] was added to each well and mixed gently and incubated for 1–2 h. in the dark at room temperature. Plates were read on a fluorescence plate reader (Synergy HT, BioTek Instruments) with excitation and emission wavelengths of 485 and 535 nm, respectively. Percent inhibition was calculated relative to growth in negative (0% inhibition) and positive controls (100% inhibition). Results are shown as mean of at least 3 replicates ± standard deviation. For each treatment, significance was determined using a one-tailed one sample t-test to determine if it significantly lowered parasite growth compared to control. IC_50_ values were estimated from a dose-response curve fitted to the normalized data using the least squares method with variable slope.

### Mass spectrometry

Active fractions from FPLC purification were further resolved by ultra-performance liquid chromatography (UPLC) and analyzed by high-resolution electrospray mass spectrometry on a Waters Acquity Xeno-G2 (UPLC-ESI-MS). Samples were dissolved in 20% DMSO and separated using a BEHC18 column (Waters, 130 Å, 1.7 μm, 2.1 × 50 mm) at 0.3 mL/min with a gradient elution of 20% to 80% aqueous acetonitrile + 0.1% formic acid. MS and MS/MS spectra were collected in positive ion mode.

### Genome curation and comparisons

Genomes were curated, and mining for secondary metabolite gene clusters was performed as described by Adamek, *et al*.^54^, with modifications. Genome sequences for nine different *Chromobacterium* species/strains were obtained from the sources listed in Table 4. Those at contig assembly level were run through MeDuSa^55^ against the sequence deposited for *C. violaceum* ATCC 12472 (NCBI RefSeq NC_005085.1), which was used as reference for further assembly. Pairwise genome average nucleotide identity (gANI) was calculated using the OrthoANIu algorithm^56^, and the resulting similarity matrix was used to generate an UPGMA tree using DendroUPGMA^57^.

**Table 4:** Sources of genome sequences of the *Chromobacterium* species/strains analyzed in this study.

Species	Strain	NCBI Identifier	Reference
*Chromobacterium sp*.	Panama	QARX00000000	This study
*Chromobacterium sp*.	W10	QARW00000000	This study
*C. aquaticum*	CC-SEYA-1	NZ_MQZY00000000.1	^69^
*C. haemolyticum*	MDA0585	NZ_JONK00000000.1	^70^
*C. piscinae*	ND17	NZ_JTGE00000000.1	^71^
*C. pseudoviolaceum*	LMG 3953	NZ_MQZX00000000.1	^72^
*C. subtsugae*	MWU2920	NZ_LCWP00000000.1	^73^
*C. vaccinii*	IIBBL 21-1	NZ_CP017707.1	^74^
*C. violaceum*	ATCC 12472	NC_005085.1	^75^

The genomic sequence obtained for *C. sp*. Panama was then uploaded to antiSMASH v. 4. (bacterial)^28,29^ and the algorithm was run together with ClusterFinder for probabilistic detection of biosynthetic gene clusters^58^. The uncurated clusters obtained were run through megaBLAST^59^ against each of the other eight *Chromobacterium* genomes and a similarity index was compounded for each query by multiplying query cover with % identity. A similarity index of 90% or above indicates that said *C. sp*. Panama gene cluster is present in the genome of that particular species; one of 30% or below indicates with confidence that the cluster is not replicated in the other genome.

### Romidepsin biosynthetic gene cluster analysis

The romidepsin biosynthetic gene cluster in *C. sp*. 968 (GenBank: EF210776.1)^35,42^ was used as reference for nucleotide sequence alignment in Geneious v5.4 (global alignment with free end gaps; cost matrix: 65% similarity) against the megaBLAST hit in each of the other *Chromobacterium spp*. genomes. Prodigal^60^ was run in each of these sequences to determine the boundaries of coding DNA sequences, and megaBLAST^59^ was used to assign their identity based on the original annotation in the 968 strain. Average pairwise sequence identity for coding sequences between the reference and other species was determined by averaging the pairwise identity values as returned by Geneious v5.4 for each of the genes in the cluster.

### *Plasmodium* gametocyte inhibition assay

Gametocyte inhibition was measured as previously described^40^. *P. falciparum* gametocyte cultures were initiated at 0.5% mixed stage parasitemia from low passage stock and cultures were maintained up to day 15 with daily media changes. At day 15 a blood smear was prepared and parasitemia was counted microscopically to calculate % mature stage V gametocytes. Gametocytes were then treated with increasing concentrations of romidepsin, 1 µM pyrvinium pamoate or 0.5% DMSO (vehicle control) for 48 hours. Gametoctyes were then washed once in drug-free serum and infectious blood meals were prepared at 0.02% gametocytemia and fed to *A. gambiae* females as described below. Data from two biological replicates (each with three technical replicates) was analyzed by the Kruskal-Wallis test by comparing number of oocysts per midgut in treatment groups to those of the control and correcting for multiple comparison by the Dunn’s test.

### *Anopheles gambiae* rearing and *Plasmodium* infection assays

*An. gambiae* Keele strain mosquitoes were maintained in the laboratory at 27 °C and 80% humidity with a 14 h day/10 h night cycle. Mosquito larvae were reared on cat food pellets and ground fish food supplement; adult mosquitoes were maintained on 10% sucrose and fed on mice anesthetized with ketamine for egg production. Female mosquitoes were infected with *P. falciparum* NF54 by allowing them to feed on stage V gametocyte cultures (0.02% gametocytemia; provided by the Johns Hopkins Malaria Institute Parasitology Core Facility) through artificial membrane feeders at 37 °C. Adult mosquitoes were starved for at least 4 h prior to feeding to guarantee robust feeding rates, and unfed mosquitoes were removed from the cohort after feeding. Mosquitoes were then incubated for a further 7–8 days at 27 °C and, to determine oocyst counts, midguts were dissected out in PBS, stained with 0.2% mercurochrome and examined using a light-contrast microscope.

To study the influence of *Chromobacterium spp*. or romidepsin on *P. falciparum* infection of *An. gambiae*, female mosquitoes were provided with roughly 10^5^ CFU/mL bacterial suspensions in 3% sucrose or romidepsin (AOBIOUS, AOB1853) in 0.5% DMSO 3% sucrose at different concentrations, respectively. Oocyst numbers were determined as described above and compared to cohorts fed on vehicle alone; median values of a combination of at least 3 replicates are shown. Significance was determined using the Kruskal-Wallis test by comparing treatment groups to the control and correcting for multiple comparison by the Dunn’s test.

### *Chromobacterium spp*. phylogenetic analysis

16 S rRNA genomic sequences from the different chromobacteria (Table 4) in addition to *C. sp*. 968 (GenBank: EF210776.1) and other related bacterial species (*Neisseria gonorrhoeae* NCTC13800; *Neisseria meningitidis* LNP21362; *Pseudomonas aeruginosa* DSM 50071) were aligned in MEGA6^61^ using ClustalW^62^. Evolutionary relationships between the sequences were inferred by the UPGMA method^63^; evolutionary distances were computed using the Maximum Composite Likelihood method^64^ and are shown in the units of the number of base substitutions per site.

### *P. falciparum* nuclear extracts and HDAC inhibition measurements

Nuclear protein extracts of *P. falciparum* NF54 were obtained as described before^65^ following the modifications by Anne Hempel from the Manuel Llinás group^66^. Briefly, *P. falciparum* NF54 cultures were pelleted (800 *g*, 5 min, low brake) and red blood cell lysis was promoted in a 0.1% PBS/saponin solution; parasites were then pelleted (800 *g*, 10 min, low brake) and lysed in 20 mM HEPES, pH 7.8, 10 mM KCl, 1 mM EDTA, 1 mM DTT, 1 mM PMSF and 1% Triton X-100. Nuclei were harvested by centrifugation at 2,500 *g* for 5 min and nuclear proteins were extracted for 30 min using 20 mM HEPES, pH 7.8, 800 mM KCl, 1 mM EDTA, 1 mM DTT, 1 mM PMSF and 1 × protease inhibitor cocktail (Sigma, P1860). Debris was removed by centrifugation (13,000 *g*, 30 min) and nuclear protein extract was kept at −80 °C in 15% glycerol until further use. HDAC inhibition by serial dilutions of romidepsin (AOBIOUS, AOB1853) or n-butanol bacterial culture supernatants in 20% DMSO was assessed using the colorimetric EpiQuik HDAC Activity/Inhibition Assay Kit (EpiGenTek, P-4002) according to the manufacturer’s protocol and using 10 µg of these *P. falciparum* nuclear protein extracts as source of *Plasmodium* HDAC enzymes.

### Data availability

The datasets generated during and/or analyzed during the current study are available from the corresponding author upon request.

## Electronic supplementary material

Supplementary Figures

Supplementary Table S1
