# Supplementary material for: Chromobacterium spp. mediate their anti-Plasmodium activity through secretion of the histone deacetylase inhibitor romidepsin
Source: Sci Rep. 2018 Apr 18;8:6176. doi: 10.1038/s41598-018-24296-0 (PMC5906607; doi:10.1038/s41598-018-24296-0)
Supplement: Supplementary file 1 — Supplementary Figures [file 41598_2018_24296_MOESM1_ESM.docx]

*Chromobacterium spp.* mediate their anti-*Plasmodium* activity through secretion of the histone deacetylase inhibitor romidepsin

Raúl G. Saraiva^1^, Callie R. Huitt-Roehl^2^, Abhai Tripathi^1^, Yi-Qiang Cheng^3^, Jürgen Bosch^4^, Craig A. Townsend^2^ and George Dimopoulos^1,*^

**SUPPLEMENTARY INFORMATION**

**
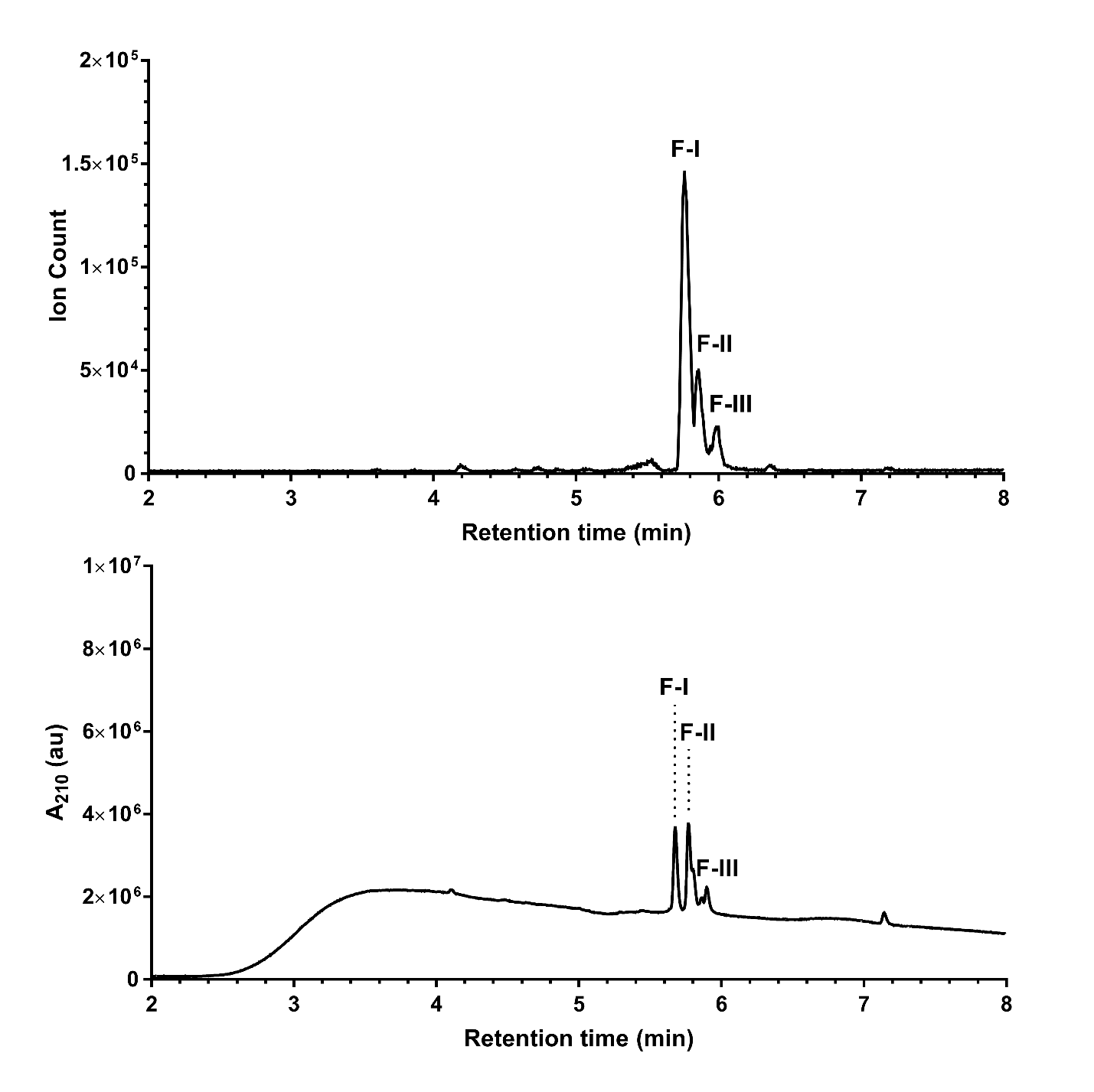
**

**Fig. S1. UPLC chromatograms of Fraction F.** (top) base peak ion chromatogram (BPI), (bottom) absorbance at 210 nm**.**

**

**Fig. S2. Predicted NRPS organization and function of the Sch 20561/2 biosynthetic cluster**. Listed in the table are *Csp_P* genes and predicted functions of encoded proteins in the putative Sch 20561/2 biosynthetic cluster. In bold are the NRPS domains that allowed prediction of a hexa(hepta)peptide core. Synthesis is hypothesized to be initiated by a (*R*)-β-hydroxylmyristoylation of the starting Dhb unit and continuing through presently indeterminent steps to the lipidated tripeptide in the first module of CspP_3681. Subsequent peptide elongation steps are assigned consistent with A domain substrate preference shown in bold above each. Final macrolactonization is well-precedented by the action of the *C*-terminal thioesterase (TE) domain to release the aglycone, Sch 20561, which is regiospecifically glycosylated to Sch 20562. adenylation domain (A), peptidyl-carrier protein (PCP), condensation domain (C), epimerization domain (E), *N*-methylation domain (nMT).

**

**Fig. S3. Tentative structural assignments of *Csp_P*-isolated Sch 20561/2**.

**Fig. S4. Survival curves of *Anopheles gambiae* upon ingestion of romidepsin at 50, 200 or 1000 µM (vehicle: 0.5% DMSO, 3% sucrose) for 24h.** *p* = 0.9736, log-rank (Mantel-Cox) test.

**Fig. S5. Survival curves of *Anopheles gambiae* (females only, males only and both) upon ingestion of *Chromobacterium sp. 968* wildtype or *∆depA* at approximately 10^6^ CFU/mL.**
